# Effect of Preoperative Nutritional Risk Screening on Postoperative Recovery in Patients with Laparoscopic-Assisted Radical Resection for Colorectal Cancer

**DOI:** 10.1155/2020/2046253

**Published:** 2020-01-06

**Authors:** Xiaolong Wang, Jianlin Wu, Sen Lei, Feng Tian, Ce Cao, Guangfeng Shi

**Affiliations:** Department of Laparoscopic General Surgery and Zibo Laparoscopic Surgery Engineering Technology Research Center, Zibo Central Hospital, Zibo, Shandong 255036, China

## Abstract

**Results:**

There were statistically significant differences in BMI, albumin, total cholesterol, and lymphocyte count between patients from the two groups (all *P* < 0.05). There was no difference in the incidence rate of postoperative complications between the two groups, but there was a statistically significant difference in the total number of complications (*P* < 0.05). There were no significant differences between the two groups regarding abdominal drainage volume, exhaust (flatus) time, hospitalization cost, morbidity, or 60 d readmission rate (all *P* > 0.05). However, patients with nutritional risk had higher postoperative blood transfusion volumes, albumin infusions, weight difference before and after surgery, and postoperative hospital stays than the nonnutritional risk group (all *P* < 0.05). Smoking, diabetes, and preoperative nutritional risk were the risk factors by the univariate and multivariate logistic regression analyses.

**Conclusions:**

The postoperative complication rate was increased, and the short-term efficacy was decreased in the preoperative nutritional risk group compared with those without nutritional risk.

## 1. Introduction

Nutritional risk refers to an existing or potential risk associated with nutritional factors that could lead to adverse clinical outcomes [1]. It not only indicates a current risk of malnutrition but also covers assessments and corrections for potential risk factors of poor nutritional status [2]. Recent studies have found that preoperative patient nutritional status influences perioperative curative effects. Therefore, evaluating nutritional status has received substantial attention from surgeons, and correcting preoperative nutritional deficiencies has become one of the most important goals in perioperative period. Currently, the common screening tools for assessing nutrition are the Nutrition Risk Screening 2002 (NRS 2002) [3], Mini Nutritional Assessment (MNA), Malnutrition Universal Screening Tool (MUST), and Nutrition Risk Index (NRI) [4]. Among them, NRS 2002 has been widely used for preoperative nutritional risk assessment for general surgery, as it can effectively predict patient clinical outcomes, which is the guiding principle for creating nutritional support programs [5]. The NRS 2002 scoring system has been recommended by the Chinese Medical Association and the European Society for Clinical Nutrition and Metabolism as one of the standards for screening inpatient nutritional risk [6].

The anatomical structure and physiological function of the gastrointestinal tract determines the risk of malnutrition and potential malnutrition in rectal cancer patients. It has been reported that preoperative malnutrition increases perioperative complications and mortality [7]. Therefore, perioperative nutritional support plays an important role in preventing postoperative complications in patients with malnutrition. In this study, colorectal cancer patients were divided into groups with or without nutritional risk by the NRS 2002 standard, and the impact of nutritional risk on postoperative recovery was retrospectively analyzed.

## 2. Methods

### 2.1. Study Design

We included 120 colorectal cancer patients (70 males/50 females) who underwent laparoscopic radical resection between January 2015 and August 2017. There were 59 colon cancer cases and 61 rectal cancer cases. The inclusion criteria were the following: (1) patients with confirmed colorectal cancer by pathological examination, (2) patients with normal communication, (3) laparoscopic radical resection of the malignancy was performed, (4) patients willing to accept nutritional risk screening, (5) no distant metastasis, and (6) no neoadjuvant radiotherapy or chemotherapy has been performed. The exclusion criteria were the following: (1) patients who received neoadjuvant radiotherapy and/or chemotherapy, (2) emergency surgery patients, and (3) those that refused to undergo nutritional risk screening. This study was approved by our institute's ethics committee.

### 2.2. NRS 2002

The NRS 2002 was performed by clinicians within 24 h of admission. NRS 2002 score equaled the sum of a patient's nutritional status score (0–3), disease severity or surgical trauma score (0–3), and age score (where >70 years = +1). An NRS 2002 cutoff of 3 was used to divide the patients, where NRS 2002 < 3 meant no nutritional risk and NRS 2002 ≥ 3 defined the group with nutritional risk.

### 2.3. Surgical Procedures and Perioperative Treatments

All patients underwent laparoscopic surgery according to routine colorectal cancer diagnosis and treatment. Laparoscopic-assisted radical resection of colorectal tumors was performed, including radical right hemicolectomy, radical transverse colectomy, radical left hemicolectomy, radical sigmoidectomy, and radical resection of rectal cancer (Dixon, Miles, and Hartmann's operation). All patients were given antibiotic prophylaxis after surgery. Routine parenteral nutrition was given, nonprotein calories were calculated at 25 kcal/kg d, and protein supplements were calculated at 0.8–1.2 g/kg d. After parenteral consumption, parenteral nutrition was gradually reduced until it discontinued.

### 2.4. Recorded Variables

The recorded preoperative parameters included smoking, diabetes mellitus, body mass index (BMI), prealbumin, transferrin, total cholesterol, blood glucose, hemoglobin, and red blood cell and lymphocyte counts. Postoperative complications were also recorded, including incision infection, pulmonary/urinary tract infection, intestinal obstruction, anastomotic bleeding, and anastomotic leakage; the total incidence of postoperative complications was also calculated. Drainage volume, first exhaust (flatus) time, first defecation time, blood transfusion volume, amount of albumin transfusion, weight difference before and after surgery, length of postsurgery hospital stay, hospitalization expenses, disease weight rate, and 60 d readmission rates were all calculated and analyzed.

### 2.5. Statistical Analysis

SPSS v23 software (IBM, Armonk, NY, USA) was used for data analyses. Measurement data were expressed as mean ± standard deviation. Counts of rows or groups that showed a normal distribution were examined by *t*-test. Counts were tested by Chi-square and Fisher's exact tests. Logistic regression analysis was used for univariate and multivariate analyses. Difference was considered statistically significant at *P* < 0.05.

## 3. Results

### 3.1. Nutritional Risk Assessments

NRS 2002 scores were <3 in 48 cases and ≥3 in 72 cases; thus, the incidence of nutritional risk was 60.00% (72/120). Among the nonnutritional risk group, men accounted for 62.50% (30/48) and women for 37.50% (18/48). The nutritional risk group was 55.55% male (40/72) and 44.45% female (32/72).

### 3.2. General Data Comparisons

General patient characteristics were compared between the two groups, and there were no significant differences in gender, age, smoking, diabetes mellitus, tumor site, transferrin, blood sugar, hemoglobin, or red blood cells (all *P* > 0.05; Table 1). Therefore, the two groups of patients were comparable. Patients in the nutritional risk group had significantly higher BMI, prealbumin, total cholesterol, and lymphocyte counts than those in the nonnutritional risk group (all *P* < 0.05; Table 1). These data indicate a poor nutritional status or potential nutritional risk in a significant percentage of this cohort.

### 3.3. Postoperative Complications

There was no significant difference in postoperative complication rates (i.e., incision infection, pulmonary/urinary tract infection, intestinal obstruction, anastomotic bleeding, and anastomotic fistula) between the two groups (all *P* > 0.05; Table 2). However, there was a difference in the total incidences of postoperative complications between the two groups. Incidences of postoperative complications were higher in the nutritional risk group than in the nonnutritional risk group (*P* < 0.05; Table 2). These results suggested that preoperative nutritional status may influence postoperative complications in colorectal cancer patients.

### 3.4. Outcomes

By investigating the postoperative recovery of patients in the two groups, we found that the postoperative hospitalization days and first defecation time of patients in the nutritional risk group were greater than those in the nonnutritional risk group (*P* < 0.05). Patients in the nutritional risk group were also significantly higher than those in the nonnutritional risk group in terms of postoperative blood transfusion volume, human albumin input, and weight differences before and after surgery (all *P* < 0.05). There were no significant differences in postoperative abdominal drainage volumes, exhaust (flatus) times, hospitalization costs, disease weight rates, or 60 d readmission rates between the two groups (all *P* > 0.05; Table 3).

### 3.5. Factors Associated with Postoperative Complications

Univariate analysis of general conditions and 14 related factors in the two groups showed that the risk of postoperative complications was associated with smoking, diabetes, and combined nutrition scores (all *P* < 0.05; Table 4). Multiple logistic regression analysis showed that smoking, diabetes, and nutritional risk were all independent risk factors for postoperative complications (odds ratios: 4.332, 8.079, and 9.835, respectively, *P* < 0.05; Table 5).

## 4. Discussion

Recent advances in laparoscopic techniques have made laparoscopic-assisted radical resection the primary operation for colorectal cancer. Laparoscopic techniques have a variety of advantages in addition to their effectiveness during surgery [8], as patients who undergo laparoscopic resection have reduced postoperative recovery times and complications compared with those treated by laparotomy [9, 10]. However, further reducing the occurrence of surgical complications after laparoscopic radical resection of colorectal cancer is still a concern; thus, searching for factors associated with surgical complications remains clinically important.

Previous studies have found that patients with preoperative nutritional risk have increased perioperative/postoperative mortality and complication rates that dramatically affect their quality of life [11]. Colorectal cancer patients may be affected by several factors that result in declined nutritional status or put them at potential nutritional risk, such as digestive dysfunction and tumor consumption [12]. Our retrospective study of 120 colorectal cancer patients found that 60.00% (72/120) had preoperative nutritional risk (NRS 2002 scores ≥ 3). Patients with nutritional risk had significantly higher postoperative complication rates, postoperative hospital stays, time of first defecation, postoperative blood transfusion volumes, albumin inputs, and weight differences before and after surgery than those without nutritional risk. Multivariate analysis showed that smoking, diabetes, and nutritional risk were independent risk factors for surgical complications.

This suggested that preoperative malnutrition may affect surgical complications and postoperative recovery. Possible reasons associated with preoperative malnutrition that may underlie the increase in postoperative complications are as follows: (1) deficient nutritional support during the perioperative period and the lack of a systematic nutritional support scheme [1]; (2) attention is only paid to postoperative nutritional deficiency, and no preoperative interventions are performed, leading to a nutritional risk that cannot be fully corrected after surgery [13]; (3) some undetected nutritional deficiencies cause increased postoperative complications and delay postoperative recovery, such as low immunity and lack of trace elements [14]; and (4) the lack of systematic preoperative nutritional support due to tight beds [15].

Based on these hypotheses, we chose cases before August 2017, because we had not previously adopted adequate preoperative nutritional support, and we tried to use a relatively consistent postsurgery nutritional support scheme, to reduce any bias caused by inconsistent perioperative nutritional support between the two groups. Our data showed that preoperative nutritional risk may increase postoperative complications and/or delay postoperative recovery. The increased postoperative complications can lead to increased postoperative hospitalization days and hospitalization costs. Evaluating the nutritional risk of colorectal cancer patients and staging preoperative nutritional interventions could improve their preoperative nutritional status, which could later reduce postoperative complications.

Univariate analysis showed that smoking, diabetes, and NRS 2000 ≥ 3 were risk factors for perioperative complications; multiple logistic regression analysis showed that they were independent risk factors for postoperative complications. However, there were some limitations in this study, such as its single-center retrospective design. In follow-up work, we will further increase the number of cases and increase the follow-up time to include curative effects; we will further study the mechanisms of how nutritional status impacts postoperative complications.

Traditional nutrition-related indicators, such as BMI, prealbumin, hemoglobin, transferrin, and lymphocyte count, are often used to evaluate the clinical nutritional status of patients. BMI is a relatively quick and simple index that measures a patient's overall nutritional status but evaluates short-term nutritional status with poor accuracy. Transferrin is the primary iron-containing protein in plasma and reflects levels of human visceral protein, which has a half-life of 7 d [16]. As an index of nutritional status, transferrin is limited and susceptible to dramatic changes following infection, decreased liver and kidney function, and anemia. Prealbumin, also known as transthyretin, is the fastest indicator of a patient's nutritional status, as its half-life is approximately 1.9 d. However, hemoglobin can only objectively reflect nutritional status from the prior month; given this delay, it cannot be used to judge short-term nutritional status [17].

In this study, BMI, prealbumin, total cholesterol, and lymphocyte count were higher in patients with nutritional risk than in those without nutritional risk, suggesting that these indicators reflect nutritional risk or potential nutritional risk; however, they could not completely replace the NRS 2002 scoring criteria. BMI, prealbumin, total cholesterol, and lymphocyte count can reflect nutritional status to a certain extent, but nutritional risk cannot be fully reflected by only taking these as the criteria. The NRS 2002 score is a screening method obtained through multicenter studies, and evidence-based medicine has shown that this scoring standard has important guiding significance for evaluating nutritional risk [18]. This standard has been applied for nutritional treatment of various malignancies, including head and neck tumors [19], pancreatic cancer [20], and liver cancer [21]. Studies have found that the rate of complications was significantly higher in patients from the nutritional risk group (according to NRS 2002 scores) than in those from the nonnutritional risk group [22]. Our results agreed with these studies, as patients in our cohort with higher NRS 2002 scores had more postoperative complications (postoperative blood transfusion volumes, albumin infusions, weight differences before and after surgery, and postoperative hospitalization days) and slower postoperative recovery than those in the nonnutritional risk group. Thus, NRS 2002 scores can be used as a risk factor for postoperative complications in patients undergoing laparoscopic-assisted radical resection for colorectal cancer.

If a quantitative nutritional risk screening methodology for colorectal cancer patients can be determined, there is the potential that perioperative nutritional support could achieve a level that would increase quality of life. However, there have been limited studies on how nutritional support can affect cure rates and/or long-term patient prognosis. Patients with nutritional risk are prone to surgical complications, and their postoperative recovery is slow, which is not favorable for subsequent adjuvant therapy [23, 24]. However, it remains a lack of multicenter large-scale clinical studies on whether nutritional risks affect long-term survival and after surgery, so this conclusion cannot be effectively or convincingly confirmed. In addition, we find that patients with preoperative malnutrition still have a higher incidence of postoperative complications (e.g., high rates of infection) after parenteral nutritional support, which may be related to the insufficient energy and protein supplementation of preoperative nutritional support. It may also be that preoperative nutritional support alters the body's metabolism, such as elevating blood sugar, which affects the incidence of complications after surgery. The reasons need further research.

In summary, the incidence of postsurgical complications in colorectal cancer patients with preoperative nutritional risk was higher than that in patients without nutritional risk. Preoperative nutritional risk was an independent risk factor for patients with surgical complications, suggesting that it could be used as an indicator of short-term outcomes. As the NRS 2002 scoring standard comprehensively and objectively reflects a patients' current nutritional status, it has proven to be of great value and can be used as a preoperative screening tool.

## Figures and Tables

**Table 1:** Clinicopathological characteristics of two groups.

	*n*	<3 (*n* = 48)	≥3 (*n* = 72)	*t*/*χ*^2^	*P*
Sex				0.571	0.450
Male	70	30 (62.50%)	40 (55.55%)		
Female	50	18 (37.50%)	32 (44.45%)		
Age				0.559	0.455
≤60 years	65	28 (58.33%)	37 (51.38%)		
>60 years	55	20 (41.67%)	35 (48.62%)		
Tumor site				1.801	0.180
Colon	59	20 (41.67%)	39 (54.17%)		
Rectum	61	28 (58.33%)	33 (45.83%)		
Smoking				1.492	0.222
Yes	47	22 (45.83%)	25 (34.72%)		
No	73	26 (54.17%)	47 (65.28%)		
Diabetes				0.067	0.796
Yes	11	4 (8.33%)	7 (9.72%)		
No	109	44 (91.67%)	65 (90.28%)		
BMI index (kg/m^2^)		22.30 ± 3.11	20.04 ± 3.92	3.509	*0.001*
Prealbumin (mg/L)		285.02 ± 37.52	233.11 ± 30.21	8.023	*0.000*
Transferrin (g/L)		2.75 ± 0.42	2.67 ± 0.12	1.285	0.204
Cholesterol (mmol/L)		4.20 ± 0.34	4.01 ± 0.51	2.449	*0.016*
Blood glucose (mmol/L)		5.50 ± 1.31	5.11 ± 0.85	1.823	0.073
Hemoglobin (g/L)		128.03 ± 31.56	120.89 ± 17.62	1.420	0.160
Erythrocyte (×10^12^/L)		4.34 ± 1.24	4.24 ± 0.82	0.492	0.624
Lymphocyte (×10^9^/L)		1.65 ± 0.42	1.43 ± 0.67	2.210	*0.029*

**Table 2:** Comparison of postoperative complications between the two groups.

	*n*	<3	≥3	*t*/*χ*^2^	*P*
Infection of incision	4	1 (2.08%)	3 (4.17%)	0.388	0.533
Lung/urinary tract infection	2	0 (0%)	2 (2.78%)	1.356	0.244
Intestinal obstruction	5	1 (2.08%)	4 (5.56%)	0.870	0.351
Anastomotic hemorrhage	4	1 (2.08%)	3 (4.17%)	0.388	0.533
Anastomotic fistula	5	1 (2.08%)	4 (5.56%)	0.870	0.351
Overall incidence (%)	20	4 (8.33%)	16 (22.22%)	4.000	*0.046*

**Table 3:** Comparison of short- and long-term postoperative recovery between the two groups.

	*n*	<3	≥3	*t*/*χ*^2^	*P*
Volume of drainage (mL)		161.53 ± 52.42	173.01 ± 72.41	0.946	0.347
Exhaust (flatus) time (h)		63.54 ± 20.06	69.42 ± 19.04	1.605	0.112
First defecation time (d)		4.56 ± 1.57	5.24 ± 0.96	2.685	*0.010*
Blood transfusion volume (mL)		204.32 ± 50.18	240.14 ± 67.41	3.349	*0.001*
Albumin infusion (g)		67.26 ± 20.08	76.32 ± 28.32	2.050	*0.043*
Weight difference before and after surgery		3.95 ± 2.21	4.95 ± 1.30	3.115	*0.002*
Postoperative hospital stay (d)		14.72 ± 4.04	16.35 ± 3.52	2.278	*0.025*
Hospitalization costs (ten thousand yuan)		5.42 ± 1.32	5.67 ± 0.95	1.131	0.261
Seriously ill rate (%)	8	2 (4.17%)	6 (8.33%)	0.804	0.370
60 d readmission rate (%)	31	10 (20.83%)	21 (29.17%)	1.044	0.307

**Table 4:** Univariate analysis of postoperative complications.

Clinical factors	*n*	Complications (*n* = 20)	No complications (*n* = 100)	*t*/*χ*^2^	*P*
Sex				0.027	0.869
Male	70	12 (60.00%)	58 (58.00%)		
Female	50	8 (40.00%)	42 (42.00%)		
Age				0.168	0.682
≤60 years	65	10 (50.00%)	55 (55.00%)		
>60 years	55	10 (50.00%)	45 (45.00%)		
Tumor site				0.807	0.369
Colon	59	8 (40.00%)	51 (51.00%)		
Rectum	61	12 (60.00%)	49 (49.00%)		
Smoking				6.722	*0.010*
Yes	47	13 (65.00%)	34 (34.00%)		
No	73	7 (35.00%)	66 (66.00%)		
Diabetes				12.510	*0.000*
Yes	11	6 (30.00%)	5 (5.00%)		
No	109	14 (70.00%)	95 (95.00%)		
Nutritional risk grouping				6.250	*0.012*
<3	48	3 (15.00%)	45 (45.00%)		
≥3	72	17 (85.00%)	55 (55.00%)		
BMI index (kg/m^2^)		21.52 ± 4.21	20.85 ± 4.28	0.648	0.523
Prealbumin (mg/L)		244.01 ± 30.17	246.27 ± 31.57	0.269	0.790
Transferrin (g/L)		2.65 ± 0.85	2.29 ± 0.51	1.829	0.082
Cholesterol (mmol/L)		4.06 ± 0.55	4.11 ± 0.72	0.351	0.728
Blood glucose (mmol/L)		5.62 ± 1.24	5.06 ± 0.95	1.911	0.069
Hemoglobin (g/L)		117.21 ± 18.32	124.34 ± 28.34	1.431	0.160
Erythrocyte (×10^12^/L)		4.26 ± 0.95	4.32 ± 1.34	0.239	0.813
Lymphocyte (×10^9^/L)		1.45 ± 0.55	1.64 ± 0.46	1.447	0.161

**Table 5:** Multivariate logistic regression analysis of postoperative complications.

Clinical factors	*B*	SE	Wald	*P*	OR (95% CI)
Smoking	1.466	.630	5.414	*.020*	4.332 (1.260-14.896)
Diabetes	2.089	.780	7.168	*.007*	8.079 (1.750-37.290)
Nutritional risk	2.286	1.071	4.559	*.033*	9.835 (1.206-80.179)

## Data Availability

The data used to support the findings of this study have not been made available because of keeping secret.
